# On Cardiac Failure and Its Treatment

**Published:** 1898-12

**Authors:** 


**On Cardiac Failure and its Treatment.** By ALEXANDER MORISON, M.D. Pp. xx., 256. London: The Rebman Publishing Company, Ltd. 1897.

Avoiding well-worn paths, Dr. Morison in some degree marks out a new line, dealing briefly with those aspects of heart failure that most writers ignore. The last half of the book is devoted to the Nauheim treatment, illustrations of the movements and details of the preparation of artificial baths being given. The writer takes a broad-minded view of the value of these methods. While he states that the judgment of those who would exalt the treatment to the position of a panacea must be disregarded, he considers that it is very justly maintained that the baths and exercises have a very wide range of usefulness. Dr. Morison has given us an interesting addition to the literature of heart disease.

